# New Developments in Myeloma Treatment and Response Assessment

**DOI:** 10.2967/jnumed.122.264972

**Published:** 2023-09

**Authors:** Françoise Kraeber-Bodéré, Bastien Jamet, Davide Bezzi, Elena Zamagni, Philippe Moreau, Cristina Nanni

**Affiliations:** 1 Médecine nucléaire, CHU Nantes, Nantes Université, Université Angers, INSERM, CNRS, CRCI2NA, F-44000, Nantes, France;; 2 Médecine nucléaire, CHU Nantes, F-44000, Nantes, France;; 3 Department of Nuclear Medicine, Alma Mater Studiorum, University of Bologna, Bologna. Italy;; 4 IRCCS Azienda Ospedaliero–Universitaria di Bologna, Istituto di Ematologia “Seràgnoli,” Bologna, Italy;; 5 Dipartimento di Scienze Mediche e Chirurgiche, Università di Bologna, Bologna, Italy;; 6 Hématologie, CHU Nantes, Nantes Université, Université Angers, INSERM, CNRS, CRCI2NA, F-44000, Nantes, France; and; 7 Nuclear Medicine, IRCCS Azienda Ospedaliero–Universitaria di Bologna, Bologna, Italy

**Keywords:** multiple myeloma, ^18^F-FDG PET/CT, MRI, radiomics, ^68^Ga-pentixafor, therapeutic evaluation

## Abstract

Recent innovative strategies have dramatically redefined the therapeutic landscape for treating multiple myeloma patients. In particular, the development and application of immunotherapy and high-dose therapy have demonstrated high response rates and have prolonged remission duration. Over the past decade, new morphologic or hybrid imaging techniques have gradually replaced conventional skeletal surveys. PET/CT using ^18^F-FDG is a powerful imaging tool for the workup at diagnosis and for therapeutic evaluation allowing medullary and extramedullary assessment. The independent negative prognostic value for progression-free and overall survival derived from baseline PET-derived parameters such as the presence of extramedullary disease or paramedullary disease, as well as the number of focal bone lesions and SUV_max_, has been reported in several large prospective studies. During therapeutic evaluation, ^18^F-FDG PET/CT is considered the reference imaging technique because it can be performed much earlier than MRI, which lacks specificity. Persistence of significant abnormal ^18^F-FDG uptake after therapy is an independent negative prognostic factor, and ^18^F-FDG PET/CT and medullary flow cytometry are complementary tools for detecting minimal residual disease before maintenance therapy. The definition of a PET metabolic complete response has recently been standardized and the interpretation criteria harmonized. The development of advanced PET analysis and radiomics using machine learning, as well as hybrid imaging with PET/MRI, offers new perspectives for multiple myeloma imaging. Most recently, innovative radiopharmaceuticals such as C-X-C chemokine receptor type 4–targeted small molecules and anti-CD38 radiolabeled antibodies have shown promising results for tumor phenotype imaging and as potential theranostics.

Multiple myeloma (MM) is a hematologic neoplasm characterized by the clonal proliferation of malignant plasma cells in the bone marrow. It is almost always preceded by an initial monoclonal gammopathy of undetermined significance, which then develops into asymptomatic, or smoldering, MM, which constitutes an intermediate clinical stage between monoclonal gammopathy of undetermined significance and MM. The definition of symptomatic MM, a clinical stage requiring treatment, was traditionally based on the presence of organ damage related to plasma cell growth as defined by the CRAB criteria (calcium elevation, renal insufficiency, anemia, and bone lesions). This definition was revised in 2014 by the International Myeloma Working Group, integrating new prognostic biomarkers with the aim of not delaying treatment of high-risk smoldering-MM–classified patients and avoiding establishment of harmful bone lesions or renal impairment (1). New biomarkers have therefore been defined that are associated with an 80% probability of progression toward positive MM CRAB criteria within 2 y, making it possible to identify patients requiring therapy; these biomarkers are a clonal bone marrow plasma cell percentage of at least 60%, an involved or uninvolved serum free light chain ratio of at least 100, and more than one bone focal lesion (FL) on MRI assessment.

Given that the presence of even an asymptomatic bone lesion must be considered a treatment criterion, the role of imaging has increased during the last few decades and modern morphologic or hybrid imaging techniques have replaced the conventional skeletal survey (2*,*^3^). It is now recommended that whole-body CT be performed as dedicated CT, or as part of PET/CT using ^18^F-FDG, when symptomatic MM diagnosis is first diagnosed and there are one or more osteolytic lesions (defining the “B” of the CRAB criteria) (4*,*^5^). Moreover, MRI detects bone abnormalities in more than 90% of patients presenting with symptomatic MM and appears to be the best procedure for evaluating painful lesions and detecting medullary compression (4). However, during the therapeutic follow-up, the performance of whole-body CT and MRI is less satisfactory because of the high frequency of false-positive images, whereas ^18^F-FDG PET appears to be more effective (4).

By detecting tumor cells or a tumor environment with high glucose consumption, ^18^F-FDG PET/CT provides additional prognostic information. MM diagnosis is associated with variable survival—short for some patients but over 10 y for others (5). These differences in survival are explained by intra- and intertumoral heterogeneity and demonstrate the potential benefits of adapting the treatment course for high-risk patients. The past decade has seen considerable advances in developing risk classifiers based on cytogenetics and gene expression profiling (6), but spatial heterogeneity can limit the sensitivity of these tests because they are based on cells obtained from a single bone marrow biopsy. Several ^18^F-FDG PET/CT characteristics could be defined as possible high-risk biomarkers and could be used to define high-risk patients at the initial diagnosis of symptomatic MM (7). ^18^F-FDG PET/CT is equally beneficial for patients with solitary plasmacytoma to detect medullary and extramedullary lesions (8*,*^9^) and has a prognostic value for patients with smoldering MM (10*,*^11^).

Other radiopharmaceuticals targeting alternative MM biomarkers have also shown promising results. These include radiolabeled choline, ^68^Ga-pentixafor targeting C-X-C chemokine receptor type 4 (CXCR4), and immuno-PET using radiolabeled monoclonal antibodies as a companion to antibody-based therapies (12). Advanced PET analysis and radiomics using machine learning and PET/MRI also appear to be promising new approaches. The goal of this review is to present new developments in MM treatment and response assessment, with a specific focus on nuclear medicine techniques.

## NEW THERAPEUTIC DEVELOPMENT

The treatment of MM has changed dramatically in the past decade with the incorporation of novel agents into therapeutic strategies. These new drugs, in various combinations, have been added to national and international clinical guidelines and have transformed our approach to the treatment of patients with MM, resulting in a significant improvement in overall survival (13*,*^14^).

With the availability of many different classes of approved agents, including alkylators, steroids, proteasome inhibitors, immunomodulatory agents, histone deacetylase inhibitors, monoclonal antibodies, and selective inhibitors of nuclear export that can be combined in double, triple, or even quadruple regimens and can be used together with or without high-dose therapy and autologous stem cell transplantation (ASCT), or in some cases as continuous treatment, the choice of the optimal strategy at diagnosis and at relapse represents a challenge for physicians (15). Moreover, contemporary next-generation immunotherapies including antibody–drug conjugates, CAR T cells, and bispecific antibodies have been approved for patients failing proteasome inhibitors, immunomodulatory agents, and CD38 monoclonal antibodies (16*,*^17^).

### Frontline Therapy

For fit patients up to the age of 70 y without comorbidities, induction followed by ASCT is the recommended treatment because of an improved progression-free survival (PFS) (13*,*^14^*,*^18^–^21^). Recently, monoclonal antibodies have been introduced in the frontline setting (22*,*^23^) as the quadruplet bortezomib, thalidomide, dexamethasone, and daratumumab (now approved by the U.S. Food and Drug Administration and the European Medicines Agency) and the quadruplet bortezomib, lenalidomide, dexamethasone, and isatuximab, both of which improve the rate of minimal residual disease (MRD) negativity (24). Quadruplet combinations before ASCT are now becoming a new standard of care. For all patients after ASCT, maintenance with lenalidomide is also considered the standard of care (25). The optimal duration of lenalidomide maintenance, approved until progression, is a matter of debate, and recent data indicate that this therapy should be proposed for at least 3 y according to tolerability (21*,*^26^*,*^27^).

For transplant-ineligible patients, outstanding outcomes have been reported when daratumumab was combined with lenalidomide and dexamethasone (28*,*^29^). This regimen is the most effective one and is approved by the U.S. Food and Drug Administration and the European Medicines Agency. Other regimens can be used as frontline therapy for transplant-ineligible patients, such as a combination of bortezomib, lenalidomide, and dexamethasone followed by lenalidomide and dexamethasone alone until progression (30*,*^31^). The quadruplet combination daratumumab plus bortezomib, melphalan, and prednisone is also approved and is associated with both PFS and overall survival benefits versus bortezomib, melphalan, and prednisone alone (32*,*^33^). Bortezomib, melphalan, and prednisone alone or lenalidomide and dexamethasone alone may be considered for patients who cannot receive the fuller regimens because of frailty (34).

### Treatment of Patients with Relapsed or Refractory MM Disease Who Have Received One Prior Line of Therapy

Overall, the most important question in most cases is whether a patient is refractory to lenalidomide. A second scenario, which will become increasingly important, is whether the patient is progressing on frontline therapies that include daratumumab (15*,*^35^–^38^). For a patient progressing on lenalidomide as part of frontline therapy, one reasonable approach is a switch in the class of agent from an immunomodulatory agent to a proteasome inhibitor. The combination of carfilzomib–dexamethasone plus anti-CD38 antibodies has recently been evaluated (39*,*^40^), with a significant improvement in PFS. On the basis of PFS data and hazard ratios, daratumumab–carfilzomib–dexamethasone and isatuximab–carfilzomib–dexamethasone, which are approved, are considered important options for the first relapse in patients with lenalidomide-refractory disease (13*,*^15^). The third best option for lenalidomide-refractory patients is the combination of pomalidomide, bortezomib, and dexamethasone (41). A significant improvement in PFS was observed with pomalidomide, bortezomib, and dexamethasone, but particularly interesting were the results in patients who had received a single previous line of treatment and were refractory to lenalidomide (42).

The most effective combination available to date in the setting of a first relapse nonrefractory to lenalidomide is daratumumab combined with lenalidomide and dexamethasone (38*,*^43^), leading to a prolonged PFS and overall survival (45.8 and 67.6 mo, respectively). Several second options for first-relapse patients with disease not refractory to lenalidomide could be proposed according to international guidelines, such as carfilzomib–dexamethasone or daratumumab plus carfilzomib–dexamethasone (13*,*^15^).

### Treatment of Patients with Relapsed or Refractory MM Who Have Received Two or More Prior Lines of Therapy

The treatment of patients who have received two or more prior lines of therapy is becoming particularly challenging. Lenalidomide and bortezomib are commonly used as part of frontline therapy or at the first relapse. Monoclonal antibodies and carfilzomib are also being increasingly used during the first 2 lines of treatment. Therefore, at the time of the second relapse, all the agents considered previously for the first relapse, but not used, can be considered (13*,*^15^*,*^44^). Some combinations approved by the U.S. Food and Drug Administration and the European Medicines Agency in this setting are isatuximab plus pomalidomide–dexamethasone (45) and daratumumab plus pomalidomide–dexamethasone

### Treatment of Triple-Class–Refractory Patients

For patients whose disease has become refractory to proteasome inhibitors, immunomodulatory agents, and anti-CD38 antibodies, the outcome is poor. Recent studies revealed that these patients have a median overall survival ranging from 6 to 12 mo (46*,*^47^). Selinexor has been evaluated in combination with dexamethasone (48) and has led to a partial response or better in 26% of patients. Consequently, in July 2019, the U.S. Food and Drug Administration granted accelerated approval to selinexor for the treatment of this subgroup of patients.

B-cell maturation antigen promotes MM pathogenesis in the bone marrow microenvironment and is a specific MM target antigen. Belantamab mafodotin is an anti–B-cell maturation antigen antibody–drug conjugate auristatin immunotoxin (49) that has achieved an overall response rate of approximately 30%–35%. Belantamab mafodotin has recently been approved by the U.S. Food and Drug Administration and the European Medicines Agency as a monotherapy for patients with relapsed or refractory MM who have received at least 4 prior therapies including an anti-CD38 monoclonal antibody, a proteasome inhibitor, and an immunomodulatory agent.

B-cell maturation antigen is also the target for 2 CAR T-cell constructs—idecabtagene vicleucel and ciltacabtagene autoleucel—that were approved in 2021 and 2022, respectively (50). These are associated with a median PFS of 8.6 mo and a median overall survival of 24.8 mo. Ciltacabtagene autoleucel has also been evaluated (51), with an overall response rate of 97.9% and 27-mo PFS and overall survival rates of 54.9% and 70.4%, respectively (52). Although the results of CAR T-cell therapies are outstanding, one important challenge is the expensive and lengthy (6–8 wk) individualized manufacturing process, which might not be feasible for patients with aggressive disease. In 2023, the access to CAR T-cell therapy remains limited. On the other hand, other immunotherapy modalities using bispecific antibodies are readily available as off-the-shelf products (16). Ongoing phase I/II clinical trials using different constructs of bispecific antibodies, with different targets on myeloma cells, are showing a favorable safety profile and high response rates in heavily pretreated patients. B-cell maturation antigen is currently the major target for bispecific antibodies, with at least 8 different compounds in preclinical or clinical development to date (53–55).

Overall, the most promising developments for triple-class–refractory patients include novel immunotherapeutic approaches, CAR T-cell therapy, and bispecific antibodies. Choosing between the 2 modalities will depend on a variety of practical considerations: efficacy, disease status, age, comorbidities, product availability, and distance from a treatment center. Moreover, early data suggest that these agents can be used sequentially and that the optimal sequencing could be CAR T cells before bispecific antibodies (56). Finally, CAR T cells and bispecific antibodies might be easily used as earlier lines of treatment to increase efficacy, and ongoing trials are already comparing these agents versus the standard of care in patients with 1–3 prior lines of therapy.

## EVALUATION OF THERAPY EFFICACY: MRD AND MODERN IMAGING

New treatment options have improved responses up to the minimum detectable level (57). Serum marker assessment and bone marrow examinations classify responders into subcategories of complete response, near-complete response, very good partial response, or partial response. However, despite an initial good clinical response, most patients relapse because of persistent disease that somehow cannot be intercepted with the standard methods.

Detection of MRD with sophisticated standardized methods at the bone marrow level, such as next-generation multiparametric flow cytometry or next-generation sequencing, with sensitivity thresholds of up to 10^−5^–10^−6^, have become over time the most important predictors of long-term outcomes and survival. They are reliable and early biomarkers of treatment effectiveness and are currently extensively applied in prospective clinical trials, sometimes as early clinical endpoints (58). A large number of studies have consistently shown that among patients achieving a complete response, those with detectable MRD have an inferior PFS and overall survival compared with those with undetectable MRD, regardless of the presence of high-risk disease features (59–61). For this reason, the latest 2016 International Myeloma Working Group consensus has introduced new response criteria, with the addition of MRD in disease assessment (62).

MRD is usually assessed in the bone marrow by means of cellular (multiparametric flow cytometry) or molecular (next-generation sequencing) methods. However, bone marrow plasma cell infiltration is often patchy, thus increasing the likelihood of a false-negative assessment by techniques that rely on a bone marrow specimen, by nature limited to a small area of the body. In addition, bone marrow evaluation does not allow one to identify extramedullary escape as a sign of metastatic spreading of the disease (63). This phenomenon is increasingly being found, as a result of prolonged survival and widespread use of functional imaging techniques, and is associated with a dismal clinical outcome, even in the novel agent era. Nonetheless, bone marrow MRD might lead to false-negative results. Despite a low rate of recurrence, MRD-negative patients can still relapse (64). Besides the patchy infiltration of bone marrow plasma cells and the presence of extramedullary disease, recent prospective studies serially monitoring patients with functional imaging and FL biopsies demonstrated that MM entails spatial heterogeneity, with possible coexistence of different disease clones, or displaying different genomic profiles in the bone marrow and in FLs (6*,*^65^). The higher the FL size, the greater the heterogeneity (65).

For a long time, imaging in myeloma has been limited to assessment of bone disease at staging or restaging and has been based on the use of a skeletal survey. This tool is useless when evaluating response to therapy, because of the low sensitivity of the technique for the limited bone healing and for soft tissues and masses. Here, functional rather than morphologic whole-body imaging techniques, such as PET/CT and MRI, which provide a comprehensive overview of the tumor burden beyond osteolytic lesions and in addition show further prognostic markers such as extramedullary disease, are the favorite tools (66). Whole-body imaging provides important complementary information about residual disease after therapy and about early relapse. Extramedullary disease sites of clonal proliferating plasma cells in the context of bone marrow MRD negativity are more frequent in patients with extramedullary disease at diagnosis (5%–10%) or with paramedullary plasmacytomas and during the relapse phases of the disease (66).

For all these reasons, the International Myeloma Working Group supports the need to assess the whole extramedullary compartment through functional imaging and bone marrow using modern biologic diagnostic tools to ensure complete tumor eradication. Although bone marrow and imaging–MRD prognostic value has been assessed, their use for clinical decision-making remains unclear. Recently, a report suggested that bone marrow MRD–based decisions during maintenance therapy could be beneficial (67), and several randomized trials are currently testing MRD maintenance strategies (68). Fewer trials are adopting an imaging–MRD adapted approach. A panel of experts published recommendations aiming to improve MRD research quality worldwide and to standardize reports (69).

## ^18^F-FDG PET/CT BEFORE THERAPY: DETECTION OF DISEASE AND PROGNOSTIC VALUE

### ^18^F-FDG PET/CT Abnormalities and Accuracy in Symptomatic MM Patients

**FIGURE 1.**
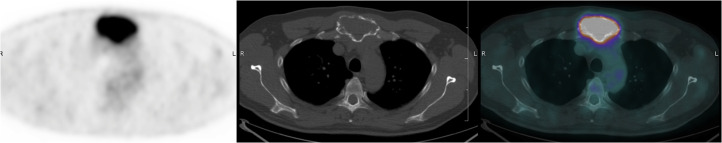
Axial PET (left), CT (middle), and PET/CT (right) images of focal osteolytic lesion with high ^18^F-FDG avidity in sternal manubrium.

**FIGURE 2.**
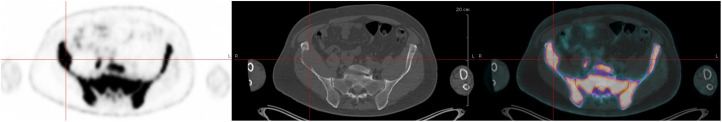
Axial PET (left), CT (middle), and PET/CT (right) images of osteolytic lesion with soft-tissue extension defining paramedullary disease in right iliac wing.

**FIGURE 3.**
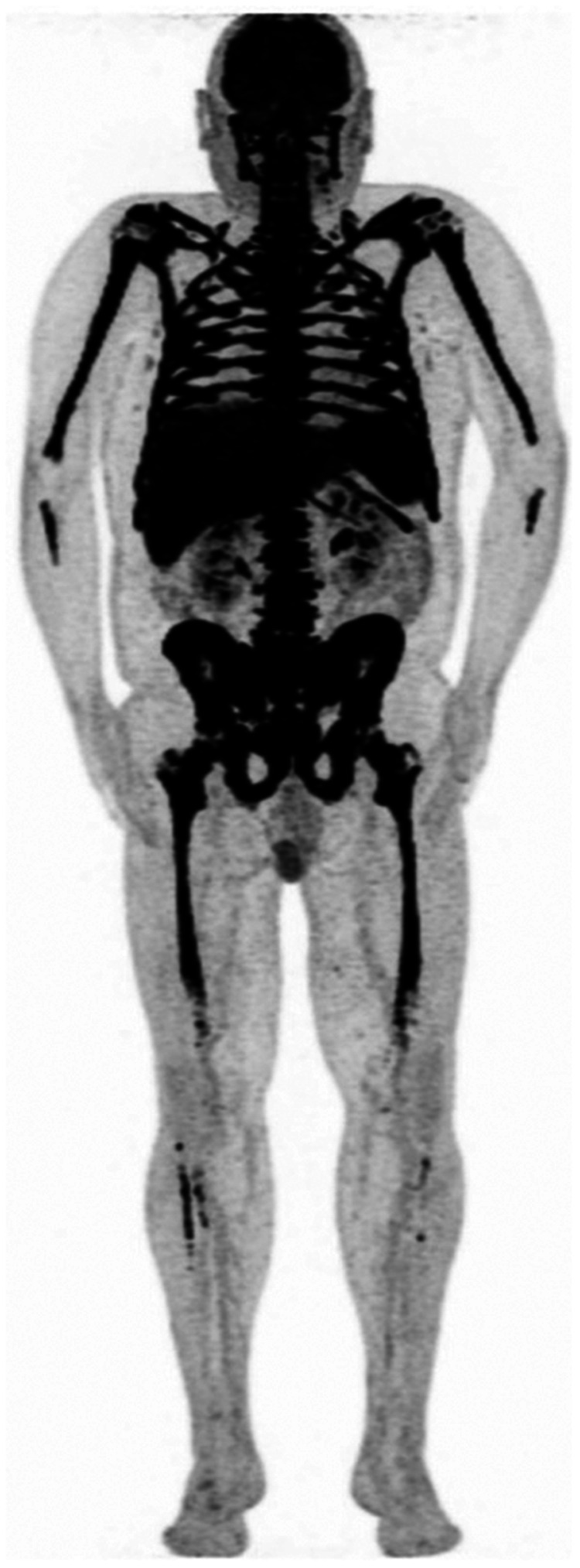
^18^F-FDG PET maximum-intensity projection showing diffuse bone marrow involvement.

**FIGURE 4.**
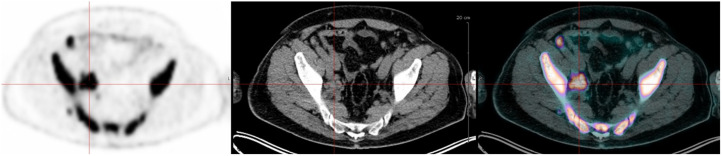
Axial PET (left), CT (middle), and PET/CT (right) images of right iliac lymph nodes with high ^18^F-FDG avidity. Biopsy favored extramedullary disease.

^18^F-FDG PET/CT has a global sensitivity of 90% for the detection of medullary disease, with a specificity varying from 70% to 100% according to several studies (70–72). Medullary abnormalities detected by PET/CT are FLs (Fig. 1), paramedullary disease (Fig. 2), and diffuse bone marrow involvement (Fig. 3) with variable glucose uptake, resulting in a variable SUV_max_ (4*,*^70^–^75^). ^18^F-FDG PET/CT also allows the detection of extramedullary disease (Fig. 4) in less than 10% of patients at initial diagnosis (76). Table 1 summarizes the elements that should be specified in ^18^F-FDG PET/CT reporting. The IMPETUS criteria have been proposed to standardize the interpretation and improve interobserver reproducibility, using a visual scale (Deauville 5-level scale) in the description of the number of FLs, extramedullary disease, and diffuse bone marrow involvement (77).

**TABLE 1.** Elements to Be Specified in ^18^F-FDG PET/CT MM Reporting

Lesion	Definition
FL	Foci of uptake above surrounding background noise on 2 successive sections with or without osteolysis on computed image, excluding benign etiologies
Extramedullary disease	Tissue invasion without contiguous bone involvement
Paramedullary disease	Soft-tissue invasion with contiguous bone involvement
Diffuse medullary involvement	Homogeneous or heterogeneous diffuse uptake of pelvic-spinal-peripheral skeleton higher than liver background
FL SUV_max_	SUV_max_ of bone FLs
^18^F-FDG PET/CT abnormality	Presence of FLs, extramedullary disease lesions, paramedullary disease lesions, or diffuse medullary involvement

In symptomatic MM, the sensitivity of ^18^F-FDG PET/CT to detect FL is greater than that of the conventional skeletal survey and comparable to or less than that of pelvic–spinal MRI (70*,*^75^*,*^78^–^81^). In the first small series of patients comparing ^18^F-FDG PET/CT and MRI, the sensitivity of ^18^F-FDG PET/CT was less than that of pelvic–spinal MRI for diffuse medullary involvement but allowed detection of additional FLs, especially outside the field of the MRI view (79). The French prospective IMAJEM study compared conventional pelvic–spinal MRI and ^18^F-FDG PET/CT at initial diagnosis and after therapy (76). In this cohort of 134 symptomatic newly diagnosed MM patients, pelvic–spinal MRI was positive in 94.7% of cases and ^18^F-FDG PET/CT in 91% of cases, revealing an equivalent detection sensitivity. Whole-body diffusion-weighted MRI, however, increases the sensitivity of MRI for FL detection, which seemed to be higher than that of ^18^F-FDG PET/CT in recent comparative (82) and simultaneous PET/MRI (83) studies.

Of patients with newly diagnosed MM, 10%–20% were ^18^F-FDG–negative, this phenomenon being associated with low expression of hexokinase-2 (84–86). These patients, however, have a better prognosis than those who have abnormal ^18^F-FDG uptake (86*,*^87^) but are not monitored after therapy by ^18^F-FDG PET/CT imaging. ^18^F-FDG PET/CT also demonstrated benefit for patients with solitary plasmacytoma, allowing detection of additional lesions with better sensitivity and specificity than conventional MRI (8). In addition, the detection of at least 2 hypermetabolic FLs by ^18^F-FDG PET/CT was reported as predictive of rapid progression of solitary plasmacytoma to symptomatic MM (9).

### Prognostic Value of ^18^F-FDG PET/CT in Baseline Evaluation of Symptomatic MM

All large, independent prospective studies conducted since 2009 have shown an independent negative prognostic impact of baseline PET-derived parameters on PFS and overall survival (88). Accordingly, a consensus statement by the International Myeloma Working Group (4) indicates that ^18^F-FDG PET/CT performed at the onset of MM is a reliable tool for prediction of prognosis. The presence of more than 3 FLs and a FL SUV_max_ of more than 4.2 have been initially suggested (89*,*^90^) as stronger negative prognostic biomarkers. Since then, different prospective studies have shown that ^18^F-FDG PET/CT normality at baseline was a protective prognostic factor (84–87). Among newly diagnosed MM patients with positive ^18^F-FDG PET/CT results, those with extramedullary disease or paramedullary disease seem to have the worst prognosis according to large prospective comparisons (76*,*^86^*,*^90^). A recent study combined transcriptomic analyses using RNA sequencing with prognostic ^18^F-FDG PET/CT biomarkers in 139 newly diagnosed MM patients included in the large prospective CASSIOPET study (85). The study confirmed that negative ^18^F-FDG PET/CT results were associated with lower expression of hexokinase-2 but also enriched for the low–bone-disease subgroup of patients. Moreover, positive ^18^F-FDG PET/CT profiles displayed 2 distinct signatures: high expression of proliferation genes and high expression of *GLUT5* and lymphocyte antigens. Paramedullary disease and the IFM15 high-risk gene expression signature were independently associated with a lower PFS, and the presence of both biomarkers defined a group of double-positive patients at a high risk of progression.

There may be scanner-specific variability in the measurement of SUV_max_, since it is higher in reconstructions with a lower Q.Clear (GE Healthcare) cutoff and more subsets in iterative reconstruction. To reduce this phenomenon, it is suggested that the standard iterative reconstruction algorithm commonly used in clinical settings (2–5 iterations with 8–28 subsets) be applied for standardizing SUV measurement.

Nevertheless, none of the studies that focused on the prognostic value of PET-derived features at baseline considered tumor heterogeneity, which could be of significant importance in MM. Recently, 2 prospective independent European randomized phase III trials (90*,*^91^) evaluated the prognostic value of baseline PET-derived features (including metabolic tumor volumes and textural features) using an innovative statistical random-survival-forest approach. These showed bone SUV_max_ as the worst negative prognostic biomarker before textural features. Volume-derived metabolic parameters such as metabolic tumor volume, total lesion glycolysis, total metabolic tumor volume, and whole-body total lesion glycolysis had less prognostic importance than others in this cohort—a finding that is discordant with previous results published by different groups. In a large cohort of patients enrolled in Total Therapy 3A, the team at Little Rock showed that whole-body total lesion glycolysis of more than 620 g and total metabolic tumor volume of more than 210 cm^3^ at baseline were independent prognostic factors for PFS and overall survival (92), but the segmentation method of bone disease used in this study is questionable. Another team’s (93) retrospective study including 185 patients with newly diagnosed MM showed that high baseline total metabolic tumor volume (>56 cm^3^) and whole-body total lesion glycolysis (>166 g) values independently predicted both worse PFS and worse overall survival, but the patients’ age was not homogeneous. Further investigations exploring the potential prognostic value of textural features in MM using artificial intelligence–based statistical approaches need to be performed.

### Prognostic Value of ^18^F-FDG PET/CT in Smoldering MM and Monoclonal Gammopathy of Undetermined Significance

^18^F-FDG PET/CT imaging has also proven useful in the setting of smoldering MM, showing prognostic value even though the latest update of the diagnostic criteria of the MM International Myeloma Working Group (1) indicate that, to consider an FL on ^18^F-FDG PET/CT as a criterion for starting therapy, osteolysis on CT is mandatory. In a first cohort of 122 smoldering-MM patients assessed by ^18^F-FDG PET/CT, the probability of progression to symptomatic MM without therapy within 2 y for patients with positive ^18^F-FDG PET/CT results (with or without osteolysis) was 75%, versus 30% for patients with negative PET results (10). Another prospective study of 120 patients (11) showed a similar rate of progression of smoldering MM to symptomatic MM at 2 y for 58% of patients with positive PET results (all without evidence of underlying osteolysis), versus 33% for patients with negative PET results. These ^18^F-FDG PET/CT results were published after the latest update of the International Myeloma Working Group’s MM definitions (1) and thus are not yet considered as a myeloma-defining event leading to the recommendation to treat these patients. Whole-body MRI is therefore the preferred imaging modality for workup of smoldering MM as recommended by the International Myeloma Working Group (94).

According to updated data on the Southeastern Minnesota cohort (with a long-term follow-up), there are adverse risk factors for progression of monoclonal gammopathy of undetermined significance to active MM, including an M-protein of 15 g/L or more and an abnormal free light chain ratio in patients with non-IgM monoclonal gammopathy of undetermined significance. Patients with 2 risk factors showed a significantly higher progression rate to MM (30% over 20 y) than did patients with no risk factors (7%) or 1 risk factor (20%) (95). Therefore, there is probably a need to image patients with a high risk for progression of monoclonal gammopathy of undetermined significance, but to date, prospective data concerning the diagnostic performance of modern functional imaging in this setting are lacking, and more specifically, there are no published data about the potential role of ^18^F-FDG PET/CT.

### Prognostic Value of ^18^F-FDG PET/CT in Patients with Relapsed or Refractory MM

^18^F-FDG PET/CT imaging is a reliable tool to detect symptomatic or paucisymptomatic bone or extramedullary MM relapse. ^18^F-FDG PET/CT discerns active from nonactive osteolytic lesions, and the targets’ SUVs are usually much higher in relapsed or refractory MM than in newly diagnosed MM. The prognostic value of ^18^F-FDG PET/CT–derived biomarkers has been highlighted several times. An absence of ^18^F-FDG–avid foci was a prognostic factor associated with a longer time to relapse and overall survival (96). The presence of more than 10 FLs also correlated with a shorter time to relapse and survival in this study. Another study reported that the presence of at least 6 FLs in the peripheral skeleton was an independent negative prognostic factor for both PFS and overall survival (97). Moreover, a high SUV_max_ (>15.9) was an independent negative prognostic factor for PFS, as was a high total lesion glycolysis for the hottest lesion (>98.1). More recently, a study evaluated the predictive value of ^18^F-FDG PET/CT parameters for relapsed or refractory MM before initiating anti-CD38 treatment. The presence of more than 3 FLs and the Multiple Myeloma International Staging System score were independently associated with inferior PFS and overall survival, allowing the identification of a population with an ultrahigh risk of relapsed or refractory MM (98).

## POTENTIAL OF ^18^F-FDG PET/CT FOR THERAPY ASSESSMENT AND MRD DETECTION

The ability to distinguish between metabolically active and inactive sites of MM makes ^18^F-FDG PET/CT an excellent tool to evaluate and monitor early and end-of-treatment response (99). Compared with morphologic classic T1- and T2-weighted MRI sequences, ^18^F-FDG PET/CT shows fewer false-positive images of persistent nonviable lesions and allows earlier and better detection of responders and prediction of outcome (2*,*^4^*,*^66^*,*^76^).

A complete normalization of ^18^F-FDG PET/CT findings after induction therapy, mainly at the premaintenance stage, is a powerful positive prognostic factor for PFS and overall survival in all large prospective studies conducted since 2009 (irrespective of the normalization used) (76*,*^90^*,*^100^–^102^), even in patients undergoing allogenic stem cell transplantation (103). This capacity to accurately demonstrate therapy response is particularly relevant in patients with nonsecretory MM, for whom the clinical assessment cannot be achieved through biochemical methods. For newly diagnosed MM patients with FL, treatment until complete PET normalization is an important therapeutic goal because their prognosis is comparable to PET-negative patients at diagnosis (104). Failure to reach complete negativity was seen for 46.4% of patients at day 7, 23.6% at the end of induction, 11.4% after transplantation, and 7.3% at premaintenance and was associated with a worse outcome. Furthermore, despite morphologic lesion stability, changes in lesion metabolism have been shown to relate to therapy efficacy, to occur in a relatively short time, and to have prognostic meaning (as early as 7 d from the start of treatment) (100*,*^104^). Bailly et al. showed that for patients with ^18^F-FDG–avid MM included in the prospective IMAJEM study after 3 cycles of RVD, the change in SUV_max_ (cutoff of 25%) appeared to be an independent prognostic factor for PFS, allowing identification of a patient subgroup with an improved median PFS (22.6 mo and not reached, respectively) (105). Zamagni et al. (90), in a cohort of 192 newly diagnosed MM patients after induction therapy and double ASCT, showed that persistence of residual ^18^F-FDG–avid FLs after induction therapy—defined as an SUV_max_ higher than 4.2—was an early predictor of shorter PFS. These findings have been confirmed in a larger patient cohort (101). Similar results were recently published in the posttransplantation setting. Kaddoura et al. showed significantly increased survival for patients reaching an ^18^F-FDG PET/CT complete response at approximately day 100 after ASCT (106). On the basis of these findings, the International Myeloma Working Group considers ^18^F-FDG PET/CT to be the standard imaging technique to monitor response to therapy in patients with MM (94), and ^18^F-FDG PET/CT has been listed as such in the MRD evaluation criteria (62).

Furthermore, the ^18^F-FDG PET/CT interpretation criteria after therapy (at premaintenance) have recently been standardized using the Deauville score (as for lymphomas) in a joint analysis of 2 prospective independent European randomized phase III trials (107). Complete metabolic response has been defined as residual uptake not exceeding the liver background activity (Deauville score, 1–3) in all initially involved bone marrow, FL, paramedullary disease, and extramedullary disease sites (Table 2).

**TABLE 2.** Interpretation Criteria for ^18^F-FDG PET/CT in MM Response to Therapy Assessment

Status	Definition
Complete metabolic response	Uptake ≤ liver activity in bone marrow sites and FLs previously involved (including extramedullary and paramedullary disease [Deauville score, 1–3])
Partial metabolic response	Decrease in number or activity of bone marrow sites/FLs present at baseline but persistence of lesions with uptake > liver activity (Deauville score, 4 or 5)
Stable metabolic disease	No significant change in bone marrow sites/FLs compared with baseline
Progressive metabolic disease	New FLs compared with baseline consistent with myeloma

Double negativity (^18^F-FDG PET/CT and multiparametric flow cytometry or next-generation sequencing in bone marrow) is a predictive surrogate for patient outcome (76*,*^108^*,*^109^). Rasche et al. showed, in a large cohort, that 12% of patients found to be MRD-negative after induction therapy still had positive FLs on ^18^F-FDG PET/CT analysis (110). Recently, a study by Böckle et al. included 102 patients with newly diagnosed MM (*n* = 57) and relapsed or refractory MM (*n* = 45), who achieved a good partial response, a complete response, or a stringent complete response by International Myeloma Working Group 2016 criteria (111). Functional imaging (^18^F-FDG PET/CT or diffusion-weighted MRI) was performed independently of bone marrow MRD results. Double-negativity rates were similar between patients with newly diagnosed MM and patients with relapsed or refractory MM, yet in the relapse setting a trend to more imaging-only positive patients was observed. Patients not achieving an optimal serologic response or MRD negativity by bone marrow and imaging were offered an individual consolidation approach: 72% of them showed a subsequent MRD conversion (51%) or deepening of serologic response levels (21%). MRD-triggered consolidation resulted in a superior PFS and overall survival comparable to a group of deep responders who achieved double-negative results after standard treatment without consolidation. In this patient population, the addition of functional imaging to bone marrow MRD assessment was helpful to tailor treatment and change prognosis.

Collectively, these results suggest that double negativity should be considered a surrogate for outcome prediction (up to deescalation strategies). However, even if a good prognostic value is demonstrated for double negativity, it is still unclear how to achieve this in patients who failed to achieve MRD negativity during standard treatment (67). Patients with double positivity or discordant results between the 2 methods (PET-positive/MRD-negative, PET-negative/MRD-positive) could represent better-stratified prognostic classes and undergo escalation or treatment modification. Finally, bispecific antibodies represent a promising and effective therapeutic approach in MM and have been recently approved by the European Medicines Agency in the relapse setting (53). Nevertheless, response monitoring using ^18^F-FDG PET/CT imaging has been shown to be challenging with therapeutic approaches involving cancer cell–T-cell interactions (i.e., CAR T cells, checkpoint inhibitor), and an ^18^F-FDG PET/CT early flare-up phenomenon could occur in MM patients receiving T-cell–engaging bispecific antibodies (112).

## PERSPECTIVES

### Non–^18^F-FDG Tracers

Many metabolic or tumor phenotype tracers have been investigated for PET imaging in MM. One of the first non–^18^F-FDG tracers used in this setting was ^11^C-methionine. ^11^C-methionine cellular uptake resembles the synthetic protein turnover by malignant cells. One advantage is that its bone marrow uptake is not influenced by anemia or systemic inflammation as for ^18^F-FDG. A prospective study of 78 patients demonstrated higher sensitivity for ^11^C-methionine PET/CT than for standard ^18^F-FDG PET/CT to detect intra- and extramedullary MM lesions, including histologic evidence of ^18^F-FDG–negative, viable disease detectable exclusively by ^11^C-methionine PET/CT (113). Similar results were recently published by Morales-Lozano et al. (114) in another prospective study (*n* = 52). ^18^F-FDG PET/CT did not detect active disease in 6 patients, whereas they were shown to be positive by ^11^C-methionine PET/CT. Additionally, ^11^C-methionine PET/CT identified a higher number of FLs than did ^18^F-FDG in more than half the patients (63%). The study also showed the prognostic value of total metabolic tumor volume and total lesion ^11^C-methionine uptake in the relapsed MM patients. Furthermore, total metabolic tumor volume p50 (median) and p75 (75th percentile) and total lesion ^11^C-methionine uptake p50 and p75 adversely impacted PFS.

Choline (either carbon- or fluorine-radiolabeled) has also been proposed as a non–^18^F-FDG tracer. At staging, this tracer has a higher positivity rate than ^18^F-FDG (115). In relapsing MM patients, ^18^F-fluorocholine PET/CT found higher numbers of lesions than ^18^F-FDG PET/CT (116). Compared with ^18^F-FDG, choline tracers can detect higher numbers of skull lesions (117). Finally, choline PET/CT was also compared with ^11^C-methionine PET/CT, with a higher detection rate for ^11^C-methionine PET/CT in approximately 40% of patients (118).

^11^C-acetate can rapidly be taken up by cells and metabolized to acetyl coenzyme A, a carbon source for fatty acid synthesis. In a group of heterogeneous MM patients, ^11^C-acetate PET/CT demonstrated better overall sensitivity and specificity than ^18^F-FDG PET/CT (119). Similar results were obtained by Lin et al., also demonstrating a positive treatment response in cases of a significant decrease in SUV_max_ (120). Recently, in a prospective single-center study (64 patients), Chen et al. (121) compared ^11^C-acetate PET/CT and ^18^F-FDG PET/CT in newly diagnosed MM. Skull deformation and lesions were more easily detected by ^11^C-acetate PET/CT. In addition, ^18^F-FDG PET/CT demonstrated a higher rate of false-positives on fractures. The presence of diffuse bone marrow uptake, more than 10 FLs, and an SUV_max_ of more than 6.0 for FLs by ^11^C-acetate PET/CT predicted a higher probability of disease progression and shorter PFS. Other tracers, such as ^18^F-sodium fluoride, ^18^F-fluciclovine, ^18^F-fluoroethyltyrosine, and ^18^F-fluorothymidine, have also been proposed; however, these are very preliminary studies (122–126). Another promising tracer is the ligand of prostate-specific membrane antigen, a characteristic biomarker for prostate cancer cells and with increased expression in the tumor vasculature. A case report indicated that ^68^Ga-prostate-specific membrane antigen–targeted ligand PET imaging can be used to visualize multiple lytic bone lesions throughout the spine, but the definitive application in MM is still unclear (127*,*^128^).

Fibroblast activation protein inhibitor PET/CT was reported to have a high specificity and affinity for targeting fibroblast activation protein. Considering the optimal biodistribution with no bone marrow uptake, this probe could be useful as a complementary imaging method to ^18^F-FDG PET/CT in some settings, especially in low ^18^F-FDG affinity and inconclusive cases (129). However, in a recent study, PET/CT with this inhibitor showed a lower activity and detection rate for MM and lymphoma than did ^18^F-FDG PET/CT (130). Finally, immuno-PET imaging with radiolabeled monoclonal antibodies or antibody fragments has potential for MRD assessment and optimization of personalized therapy. ^64^Cu-DOTA- or ^89^Zr-desferrioxamine-daratumumab might be useful (131*,*^132^), as well as anti-CD138 targeted imaging (133*,*^134^). Anti-CD38 immuno-PET could be used to identify MM patients who would benefit from daratumumab and thus predict the effectiveness of treatment. Further validation of all these agents in larger patient cohorts and clinical trials is important.

^68^Ga-pentixafor targeting CXCR4 has also been evaluated in MM patients. CXCR4 expression frequently occurs in advanced MM and probably represents a negative prognostic factor (135*,*^136^). This tracer could be relevant for prognostic imaging or a theranostic approach before radioligand therapy using pentixather targeting CXCR4 labeled both with ^90^Y and with ^177^Lu. Indeed, ^68^Ga-pentixafor seemed to be a prognostic stratifier in terms of overall survival in a population of 35 patients affected by relapsed or refractory MM (135). The prognostic characteristics turned out to be positivity versus negativity of the scan, presence of ^68^Ga-pentixafor–positive extramedullary disease, and positive appendicular bone marrow. Nevertheless, in this population ^18^F-FDG PET/CT was able to detect significantly more localizations than ^68^Ga-pentixafor. Another study, conducted on 34 MM patients at diagnosis, found that the target-to-background ratio of ^68^Ga-pentixafor was higher in 27 of the included patients, and the bone stage of the disease increased from I to II in 1 patient, I to III in 5 patients, and II to III in 3 patients as compared with ^18^F-FDG PET/CT (137). In only one patient did ^68^Ga-pentixafor detect fewer FLs than ^18^F-FDG PET/CT. The excellent target-to-background ratio of ^68^Ga-pentixafor imaging (compared with ^18^F-FDG PET/CT) in patients with CXCR4 overexpression has been confirmed in the preliminary results of another ongoing prospective study (138). Although very preliminary, these studies are fundamental to correctly guide future CXCR4-targeted theranostic trials. A preliminary study using pentixather labeled with both ^90^Y and ^177^Lu has also been reported (139). Three patients in a late stage of disease were treated in the context of compassionate use, and one of them achieved a complete response at both skeletal and extraskeletal localizations, making this compound interesting for future applications. Finally, although unpublished, compounds based on fibroblast activation protein inhibitor and prostate-specific membrane antigen also warrant attention as potential theranostics in the near future.

### Radiomics and Machine Learning

Tumor heterogeneity, as described at the cellular level, could probably be partly captured through medical image analysis, especially using PET-based images. This type of image analysis, often referred to as radiomics, has gained significant interest in the past few years, with several studies underscoring the potential of textural features. The high number of features extracted from a radiomics approach advocates the use of adapted statistical analysis given the highly dimensional nature of the problem and the associated risk of overfitting with low-complexity models (91). In this respect, a random-survival-forest approach outperformed more conventional approaches for prognosis purposes (140).

The potential prognostic value of ^18^F-FDG PET–derived radiomics at baseline in newly diagnosed MM was explored for the first time recently in a combined analysis of 2 independent prospective European phase III trials using a random-survival-forest approach (91). Among all image features and clinical and histopathologic parameters collected, radiomics were not retained in the final prognosis model based on a random survival forest and set by only 3 features but belonged to the most predictive variables. Further investigations exploring the potential prognostic value of textural features in MM using the random-survival-forest approach are going to begin soon in a larger cohort of patients included in the multicenter international CASSIOPET study (86).

Furthermore, Tagliafico et al. showed that a radiomics approach could improve radiologic evaluation of MM on CT (141). Using a small retrospective cohort, Schenone et al. reported that artificial intelligence and radiomics’ features prognostically stratified MM patients (142). Park et al. reported that machine learning provided an accurate and reliable diagnosis of diffuse bone marrow infiltration in MM patients (143). Jin et al. applied to ^18^F-FDG PET/CT a radiomics model that classified MM and bone metastases (144). Liu et al. demonstrated that a logistic regression–based machine learning method may be superior to other methods for assessing high-risk cytogenetic status in MM (145). Other radiomics methods were applied to MR images and were coupled to molecular and clinical information (146).

Many studies are retrospective, and when there is a small pool of patients, data mutability is a main problem for using such systems in hematology. It is currently unknown how these procedures would operate with inter- and intralaboratory variability. Moreover, current artificial intelligence techniques are not transparent in their elaboration processes; their interlocutors might not know how artificial intelligence techniques have reached a given conclusion: this could produce trust issues, especially when critical choices need to be based on these conclusions. For this reason alone, the application of artificial intelligence in clinical settings is in a preliminary phase (146*,*^147^).

### Whole-Body Multiparametric Functional MRI

^18^F-FDG PET/CT and whole-body MRI are both included in the updated International Myeloma Working Group definition of MM criteria and are used to evaluate the number of FLs and the presence of bone marrow involvement as surrogates of disease burden (94). The prospective IMAJEM study concluded that there was no difference in the detection of bone involvement at baseline MM between conventional pelvic–spinal MRI (without whole-body diffusion-weighted imaging) and whole-body ^18^F-FDG PET/CT on a patient-based analysis (76).

**FIGURE 5.**
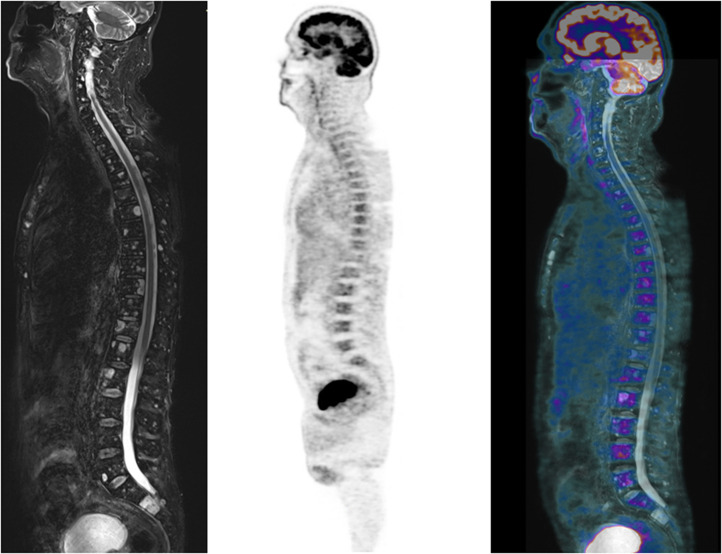
Sagittal T2-weighted short-τ inversion recovery MR (left), PET (middle), and PET/MR (right) images of disseminated FLs of spine. There is no evidence of increased ^18^F-FDG avidity corresponding to MRI findings.

**FIGURE 6.**
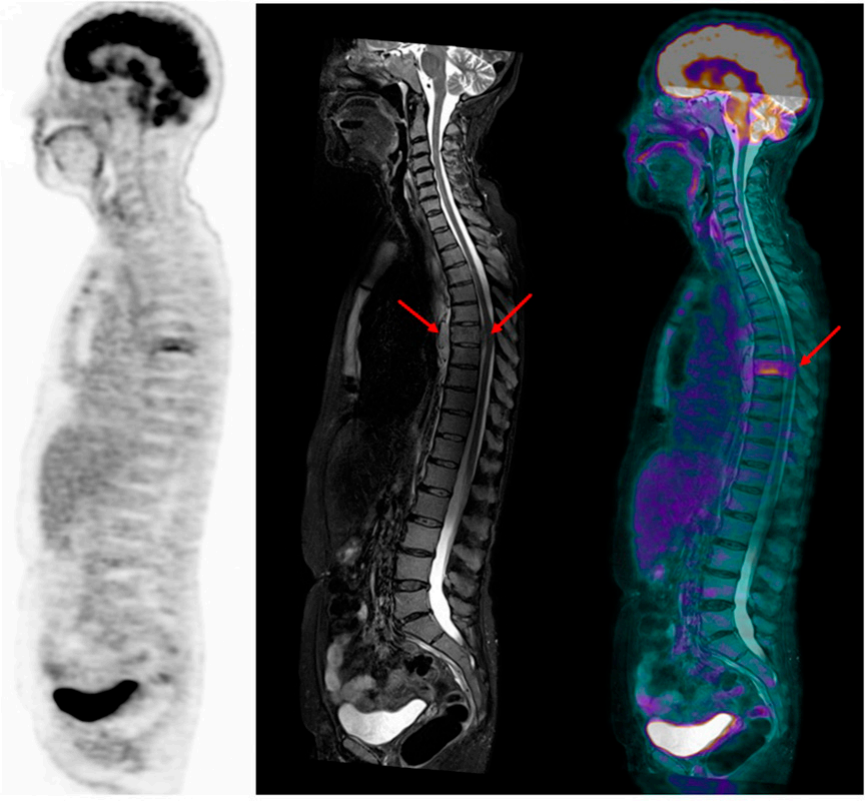
Sagittal PET (left), T2-weighted short-τ inversion recovery MR (middle), and PET/MR (right) images. Homogeneous diffuse bone marrow involvement is seen on MRI (diffuse homogeneous increased signal), with added FL in T7 body (with ^18^F-FDG avidity). Anterior and posterior soft-tissue extension (arrows) defines paramedullary disease and leads to cord compression.

The excellent image contrast between normal and diseased bone marrow by diffusion-weighted MRI leads to superior FL detection compared with conventional morphologic (included short-τ inversion recovery) and contrast-enhanced MRI sequences (148–150) and therefore is increasingly being used for MM work-up (151*,*^152^). The whole-body MRI protocol in MM should include axial T1- or T2-weighted turbo spin echo short-τ inversion recovery sequences, axial diffusion-weighted imaging with 2 b values (50 and 800 s/mm^2^) and apparent diffusion coefficient (ADC) calculation with 3-dimensional MIP reconstructions of the highest b-value images, and whole-spine sagittal T1-weighted Dixon/T2-weighted short-τ inversion recovery turbo spin echo weighted sequences. The sensitivity of MRI for FL detection including this sequence seemed to be higher than that of ^18^F-FDG PET/CT in recent comparative (82*,*^153^) and simultaneous PET/MRI (83) studies (Figs. 5 and 6).

Imaging with more than one b value allows automated calculation of the ADC for each voxel in the image, and a quantitative map can be produced. A tumor—with tightly packed cells—therefore appears as an area of restricted water diffusion and high signal on a source diffusion image and of low value on an ADC map, and response to therapy induces decreased cellularity, thereby diminishing the signal at a high b value and increasing the ADC values. Therefore, diffusion-weighted imaging theoretically allows therapy assessment. In a large cohort, Weinhold’s team (110) highlighted more patients with residual FL on diffusion-weighted imaging than on ^18^F-FDG PET/CT imaging at the onset of complete response after first-line or second-line therapy. There were patients with FLs in the PET-only analysis too, suggesting that the 2 techniques are complementary. Because the ADC cutoff value for FL positivity definition after therapy remains undetermined, the specificity of this sequence remains unclear. However, in this study, residual FLs—detectable in 24% of first-line patients—were associated with shorter PFS. Furthermore, the PFS of patients with residual FLs that were detectable only using whole-body diffusion-weighted imaging was not significantly different from that of patients with residual PET-positive FLs (median PFS, 3.4 vs. 3.0 y). In another recent study (154*,*^155^), sustained MRD negativity assessed by diffusion-weighted imaging during maintenance therapy 1 y after ASCT had strong predictive relevance for survival in newly diagnosed MM patients.

Another study (156) showed that mean ADC increased in all but 1 of 20 patients who responded to treatment, whereas ADC decreased in all 5 patients who did not respond to treatment. ADC measurement was repeatable. ADC changes after therapy are included in the Myeloma Response Assessment and Diagnosis System MRI guidelines (152) to help define the response assessment category. The “highly likely to be responding” patient category includes previously evident lesions showing an increase in ADC from less than 1,400 μm^2^/s to more than 1,400 μm^2^/s and less than a 40% increase in ADC from baseline with a corresponding decrease in normalized high b-value signal intensity and morphologic findings consistent with stable or responding disease. We have to keep in mind that ADC values are influenced by many parameters, including the choice of b values, the diffusion time achievable with diffusion sequences, and both patient- and technique-related features (e.g., magnetic field strength and coils).

Another MRI-derived feature that could help for response to therapy assessment, especially in ^18^F-FDG PET/CT–negative patients, is the fat fraction of FLs. In a study by Latifoltojar et al. (157), among different quantitative biomarkers extracted from MRI, signal fat fraction and ADC significantly increased in responders but not in nonresponders. FL fat fraction was the best discriminator of treatment response, and bone fat fraction repeatability was better than bone ADC repeatability.

Finally, new hybrid simultaneous PET/MRI has emerged recently, and these devices substitute MRI (coupled with PET) for the usual CT scan. For MM, the theoretic and practical benefit of performing single-shot simultaneous ^18^F-FDG PET/MRI is high, but to date there are scarce data published. A preliminary retrospective study showed that whole-body PET/MRI provided optimal diagnostic performance (83). However, the study could not compare PET/MRI and PET/CT. Further trials assessing the diagnostic and prognostic performance of PET/MRI are needed.

## CONCLUSION

^18^F-FDG PET/CT represents a powerful tool for the detection of medullary and extramedullary disease at the diagnosis of symptomatic MM, with a negative prognostic value for a high medullary SUV_max_ and for the presence of extramedullary disease and paramedullary disease. ^18^F-FDG PET/CT is considered the reference imaging technique for therapy assessment, evaluation being possible earlier than for conventional MRI. The negativity of pre-ASCT ^18^F-FDG PET/CT is a favorable prognostic factor, and the positivity of ^18^F-FDG PET/CT after ASCT, especially in patients with a complete biologic response, is an independent negative prognostic factor. Negative finding on ^18^F-FDG PET/CT, normal on intramedullary flow cytometry, and a normal ratio of serum free light chains would allow definition of an optimal complete response (eradication of monoclonal plasma cells in all compartments). Ongoing prospective trials will try to confirm the complementary role of functional imaging with molecular techniques for the detection of MRD inside and outside the bone marrow at relapse. ^18^F-FDG PET/CT is the best imaging technique to differentiate active disease from morphologic scars and remodeling. Other PET tracers and PET combined with MRI may also show benefit, especially in patients with false-negative ^18^F-FDG findings, but should be evaluated in prospective clinical trials. Radiomics and machine learning methods may improve the prognostic value of PET images.
